# IFNγ and iNOS-Mediated Alterations in the Bone Marrow and Thymus and Its Impact on *Mycobacterium avium*-Induced Thymic Atrophy

**DOI:** 10.3389/fimmu.2021.696415

**Published:** 2021-12-20

**Authors:** Palmira Barreira-Silva, Rita Melo-Miranda, Claudia Nobrega, Susana Roque, Cláudia Serre-Miranda, Margarida Borges, Gisela Armada, Daniela de Sá Calçada, Samuel M. Behar, Rui Appelberg, Margarida Correia-Neves

**Affiliations:** ^1^ Life and Health Sciences Research Institute (ICVS), School of Medicine, University of Minho, Braga, Portugal; ^2^ Life and Health Sciences Research Institute/Biomaterials, Biodegradables and Biomimetics Research Group (ICVS/3B’s), PT Government Associate Laboratory, Braga, Portugal; ^3^ Research Unit on Applied Molecular Biosciences (UCIBIO)/Rede de Química e Tecnologia (REQUINTE), Departamento de Ciências Biológicas, Faculdade de Farmácia, Universidade do Porto, Porto, Portugal; ^4^ Department of Microbiology and Physiological Systems, University of Massachusetts Medical School, Worcester, MA, United States; ^5^ Instituto de Investigação e Inovação em Saúde (i3S), Instituto de Investigação e Inovação em Saúde, Universidade do Porto, Porto, Portugal; ^6^ IBMC-Instituto de Biologia Molecular e Celular, Universidade do Porto, Porto, Portugal; ^7^ ICBAS-Instituto de Ciências Biomédicas Abel Salazar, Universidade do Porto, Porto, Portugal; ^8^ Division of Infectious Diseases, Department of Medicine Solna, Karolinska Institutet, Stockholm, Sweden

**Keywords:** thymus premature atrophy, *Mycobacterium avium* infection, IFN gamma, nitric oxide, BM T cell precursors, thymocyte differentiation

## Abstract

Disseminated infection with the high virulence strain of *Mycobacterium avium* 25291 leads to progressive thymic atrophy. We previously showed that *M. avium*-induced thymic atrophy results from increased glucocorticoid levels that synergize with nitric oxide (NO) produced by interferon gamma (IFNγ) activated macrophages. Where and how these mediators act is not understood. We hypothesized that IFNγ and NO promote thymic atrophy through their effects on bone marrow (BM) T cell precursors and T cell differentiation in the thymus. We show that *M. avium* infection cause a reduction in the percentage and number of common lymphoid progenitors (CLP). Additionally, BM precursors from infected mice show an overall impaired ability to reconstitute thymi of RAGKO mice, in part due to IFNγ. Thymi from infected mice present an IFNγ and NO-driven inflammation. When transplanted under the kidney capsule of uninfected mice, thymi from infected mice are unable to sustain T cell differentiation. Finally, we observed increased thymocyte death *via* apoptosis after infection, independent of both IFNγ and iNOS; and a decrease on active caspase-3 positive thymocytes, which is not observed in the absence of iNOS expression. Together our data suggests that *M. avium*-induced thymic atrophy results from a combination of defects mediated by IFNγ and NO, including alterations in the BM T cell precursors, the thymic structure and the thymocyte differentiation.

## Introduction

T cell progenitors migrate from the bone marrow (BM) to the thymus, where they encounter a specialized environment that promotes their differentiation into new T cells. The thymus undergoes physiological involution with aging (1, 2). This, together with reports from late 1990s showing that removal of the thymus at an early age seemed to have little consequence on the normal T cell repertoire of adults, led to the idea that the thymus was only needed for T cell production early in life (3). However, further investigation showed that thymectomy early in life leads to premature immune aging (4). Importantly, the amount of functional thymic tissue correlates with the reconstitution of the peripheral T cell repertoire after severe lymphopenia, as is the case of patients with acquired immunodeficiency syndrome (AIDS) under antiretroviral therapy, or in patients with cancer after BM transplant (5, 6). These data highlight the importance of understanding premature thymic atrophy, which can affect the overall immune health of the individual.

A variety of pathogens target the thymus and induce premature thymic atrophy in humans, such as the Human Immunodeficiency Virus (HIV), and experimental animal models, such as the bacterium *Mycobacterium avium*, the fungus *Paracoccidioids brasiliensis*, the parasite *Tripanosoma cruzi*, and the virus Murine Leukemia Virus (MuLV), among others (7, 8). Although the thymus is a target for infection with different mycobacteria (9), *M. avium*-induced thymic atrophy has been shown to be strain-dependent (10). For example, premature thymic atrophy develops after infection with the high virulence strain *M. avium subspecies avium* strain 25291, but no signs of atrophy develop even after several months of chronic infection with the low virulence strain 2447 (10, 11).

*M. avium* infection-induced thymic atrophy arises from a synergy between glucocorticoids (GC) and nitric oxide (NO) produced by interferon gamma (IFNγ) activated macrophages (Mϕ), since mice lacking IFNγ, iNOS or the activation of Mϕ by IFNγ show no infection-induced thymic atrophy (10). However, where and how these mediators act to lead to *M. avium* infection-induced thymic atrophy is unknown.

Different alterations in the thymus occur during premature thymic atrophy observed in different mouse models of infection (7, 8, 12). Mechanisms that have been defined include: (i) thymic structural changes (13, 14); (ii) reduction in thymocyte proliferation (15); (iii) increased thymocyte cell death (16–19); and, (iv) increased export of immature thymocytes to the periphery (20–22). A frequently described mechanism is GC overproduction that causes excessive death of thymocytes, mostly at the double positive (DP) stage (23, 24). Additionally, IFNγ and NO can also induce thymocyte cell death, either independently or by synergizing with GC (16, 25–28).

A possible mechanism of premature thymic atrophy, which has been seldom explored, is alterations of T cell precursors in the BM, and could include: (i) decreased production; (ii) arrest in the BM and consequent decreased migration and/or (iii) loss of ability to populate the thymus. In fact, infection-induced alterations of the BM have been abundantly documented following infection by bacteria including *Mycobacterium tuberculosis* (29), *M. avium* (30), Group A *Streptococcus* (31) and *Escheriachia coli* (32); viruses including the HIV (33), Pneumovirus (34) and Vaccinia virus (35); and parasites such as *Plasmodium chabaudi* (36), *Plasmodium berghei* (37) and *Trypanosoma brucei* (38). IFNγ is the major mediator produced during some of these infections that promotes alterations in BM cells (30, 31, 34, 36), *via* the expansion of LSK (Lineage^-^ Sca1^+^ cKit^+^) cells and the increased differentiation of cells from the myeloid lineage (39). Other molecules such as tumor necrosis factor (TNF), NO and type I IFN have also been associated with BM alterations during infection (34, 38, 40). However, except for two reports showing that the reduction of BM precursors is associated with sepsis-induced and *T. cruzi* infection-induced thymic atrophy (41, 42), we are not aware of other reports that associate alterations in the BM with infection-induced thymic atrophy.

We previously showed that *M. avium* strain 25291 infected wild type (WT) mice have a reduction in the number of early thymic precursors (ETP) (10), which is the most immature cell population in the thymus. Additionally, mice that lack iNOS expression (iNOSKO mice) are resistant to infection-induced thymic atrophy, but have also reduced numbers of ETP (10). While these data indicate that alterations in the BM T cell precursors may not be sufficient to cause premature thymic atrophy, it could be an important step in the process. Here, we took advantage of KO mouse strains, cell transfer and thymic transplant models to study how alterations mediated by IFNγ and/or iNOS affecting the thymic environment and/or the BM contribute to premature thymic atrophy during *M. avium* infection.

## Materials and Methods

### Mice and Infection

C57BL/6 WT mice were purchased from Charles River Laboratories (Barcelona, Spain) or bred at the Life and Health Sciences Research Institute (ICVS - School of Medicine, University of Minho, Braga, Portugal) from a breeding pair purchased from Charles River Laboratories. B6.SJL-*Ptprca Pepcb*/BoyJ (WT CD45.1) and IFNγ–KO C57BL/6 mice were bred at ICVS from a breeding pair purchased from The Jackson Laboratory (Bar Harbor, ME, USA). Transgenic mice with a selective impairment on IFNγ signaling in CD68^+^ cells (MIIG) (43) were bred at the Institute for Molecular and Cell Biology (University of Porto, Porto, Portugal) from a breeding pair provided by the Cincinnati Children’s Hospital Medical Center and the University of Cincinnati College of Medicine (Cincinnati, OH, USA). iNOS-deficient C57BL/6 (iNOSKO) (44) mice were bred at ICVS after back-crossing the original strain (kindly provided by Drs. J. Mudgett, J.D. MacMicking, and C. Nathan, Cornell University, New York, NY, USA) onto a C57BL/6 background for seven generations, or purchased from The Jackson Laboratory. B6(Cg)-*Rag2^tm1.^* ^1Cgn^ (RAGKO) mice were bred at ICVS from a breeding pair purchased from Instituto Gulbenkien da Ciência (Oeiras, Portugal) or from The Jackson Laboratory. iNOS and Rag2-double deficient (iNOS.RAG.2KO) mice were obtained, at ICVS, after crossing F1 resulting from the cross of iNOSKO and RAGKO mice.

Eight- to ten-week-old female mice were infected intravenously (i.v.) through a lateral tail vein with 10^6^ colony forming units (CFU) of the *M. avium* strain ATCC 25291 SmT (obtained from the American Type Culture Collection, Manassas, VA, USA) or the strain 2447 (provided by Dr. F. Portaels, Institute of Tropical Medicine, Antwerp, Belgium). Bacterial inocula preparation and bacterial load quantification in the organs was performed as previously described (11, 45). No signs of major distress were observed for the first 2 months upon infection, though some animals showed signs of deterioration of body condition in a non-synchronous way. To avoid excessive and unnecessary suffering of animals, humane endpoints were applied, and mice were euthanized when reaching 25% weight loss, relatively to the highest weigh reached.

Mice euthanasia was performed through controlled CO_2_ inhalation or an overdose of ketamine (150 mg/kg) and medetomidine (2 mg/kg) injected intraperitoneally (i.p.), followed by lethal blood collection (performed after confirmation of anesthesia) and thoracotomy.

All experiments were performed in accordance with the recommendations of the European Convention for the Protection of Vertebrate Animals Used for Experimental and Other Scientific Purposes (ETS 123) and 86/609/EEC Directive and Portuguese rules (DL 129/92). The animal facility and people directly involved in animal experiments were certified by the Portuguese regulatory entity - *Direção Geral de Alimentação e Veterinária* (DGAV); the animal experimental protocols were approved by DGAV (# 015584), or by the Institutional Animal Care and Use Committee at the University of Massachusetts Medical School (Animal Welfare A3306-01), using the recommendations from the Guide for the Care and Use of Laboratory Animals of the National Institutes of Health and the Office of Laboratory Animal Welfare.

### Single Cell Suspensions

Spleens, thymi and BM were collected and processed individually. Single cell suspensions were obtained from thymi and spleens by gentle mechanical dissociation in complete DMEM (cDMEM - DMEM supplemented with 10% heat-inactivated FBS, 10 mM HEPES, 1 mM sodium pyruvate, 2 mM L-glutamine, 50 mg/ml streptomycin, and 50 U/ml penicillin, all from Invitrogen Life Technologies); and from femurs by gentle flush of the BM with cDMEM. Red blood cells were lysed using a hemolytic solution (155 mM NH_4_Cl, 10 mM KHCO_3_, pH 7.2) for 4 min at room temperature, and cells were re-suspended in cDMEM. The number of viable cells was counted by trypan blue exclusion using a hemocytometer.

### Flow Cytometry

One million cells were stained per panel for flow cytometry analysis. For BM analysis, cells were stained with FITC-conjugated anti-lineage markers [anti-CD3 (145-2C11), anti-CD4 (RM4-5), anti-CD8 (53-6.7), anti-CD19 (6D5), anti-B220 (RA3-6B2), anti-CD11b (M1/70), anti-CD11c (N418), anti-NK1.1 (PK136), anti-Gr1 (RB6-8C5), anti-TER119 (TER119)], PE-conjugated anti-cKit (2B8), PerCP-Cy5.5-conjugated anti-Sca1 (D7), PE-Cy7–conjugated anti-IL7Rα (A7R34), APC-conjugated anti-Flt3 (A2F10), APC-Cy7-conjugated anti-CD48 (HM48-1) and BV421-conjugated anti-CD150 (TC15-12F12.2). For thymocyte analysis, cells were labeled with distinct combinations of the following antibodies: FITC, PerCP-Cy5.5, BV510 or V500-conjugated anti-CD8 (53-6.7), PerCP-Cy5.5-conjugated anti-CD24 (M1/69), PE or APC-conjugated anti-CD3 (145-2C11), PE-Cy7 or APC-Cy7-conjugated anti-CD44 (IM7), APC-Cy7 or V450-conjugated anti-CD4 (RM4-5), Alexa647-conjugated anti-active caspase-3 (C92-605) and APC-conjugated Annexin V. For live/dead cell analysis, propidium iodide (PI; Sigma-Aldrich, Germany) was added at a final concentration of 1 mg/ml, 15 min before acquisition on the flow cytometer. For splenocyte analysis, cells were labeled with the following antibodies: FITC-conjugated anti-CD11b (M1/70), APC-conjugated anti-CD3 (145-2C11), APC-Cy7-conjugated anti-CD19 (6D5), V450-conjugated anti-CD4 (RM4-5) and V500-conjugated anti-CD8 (53-6.7). All antibodies were purchased from BioLegend (San Diego, CA, USA) except the V450-conjugated anti-CD4 (RM4-5), the V500-conjugated anti-CD8 (53-6.7) and the Alexa647-conjugated anti-active caspase-3 (C92-605), which were obtained from BD Biosciences (San Jose, CA, USA). Acquisition was performed on a LSRII flow cytometer (equipped with 3 lasers: blue - 5 detectors, red - 2 detectors and violet - 6 detectors) using BD FACSDiva software v6.0 (Becton and Dickinson, NJ, USA), or on a MACSQuant flow cytometer (Miltenyi Biotec, Germany; equipped with 3 lasers: blue - 5 detectors, red - 2 detectors and violet - 2 detectors). Data were analyzed using FlowJo 10.7.1 (BD Biosciences).

### Thymic Transplant

Thymi were aseptically removed from uninfected RAGKO or from WT mice infected for 70 days with *M. avium* 25291. Thymi were maintained in cDMEM for no longer than 20 min until being transplanted under the kidney capsule of WT CD45.1 mice (anesthetized with 200 mg/Kg xylazine hydrochloride and 200 mg/Kg ketamine hydrochloride, administered i.v.). One thymic lobe from uninfected RAGKO mice and one thymic lobe from infected WT mice were transplanted to the same WT CD45.1 mouse (one on each kidney). Mice were euthanized 4 weeks after transplant, and the transplanted thymi analyzed by flow cytometry.

### BM Adoptive Transfer

Single-cell suspensions of pools of BM cells were prepared from uninfected or from 70 days *M. avium* 25291 infected WT or IFNγKO mice. BM progenitor cells were purified from each suspension using the Lineage Cell Depletion Mouse Kit microbeads (Miltenyi Biotec). Magnetic separation was performed with an autoMACS separator (Miltenyi Biotec). After purification, viable cells were counted by trypan blue exclusion using a hemocytometer; purity was confirmed by flow cytometry stain using FITC-conjugated anti-lineage markers and PE-conjugated anti-cKit. Cells (1-1.5 x 10^6^) were transferred i.v. to RAGKO or iNOS.RAG.2KO mice treated with Busulfan (0.6 mg/mouse) administered i.p. 24 h before. Recipient mice received prophylactic antibiotic treatment *ad libitum* [2,5% of Bactrim (40 mg trimethoprim and 200 mg sulfametoxazol) in drinking water] for 5 days (treatment finished 2 days before BM transfer). Mice were euthanized 4 weeks after cell transfer and the recipient thymus and spleen were analyzed by flow cytometry.

### Measurement of Corticosterone Serum Levels

To obtain the serum concentration of basal corticosterone levels, blood samples were collected at 9 am (1 h after lights are on at the animal facility) from a venous incision at the tip of the tail during a period not exceeding 2 min for each mouse (to avoid corticosterone sera level increase due to handling). Sera were isolated by centrifugation and stored at -80 °C. Corticosterone levels were determined using Corticosterone ELISA kit (ENZO life sciences, Inc., NY, USA) according with manufacturer’s instructions.

### Real Time-Quantitative PCR Analysis

Total RNA was extracted from 1 thymic lobe (except for 70 dpi, where the whole thymus was used due to severe atrophy) and from BM cells recovered from two femurs per mouse, using TRIzol™ Reagent (Invitrogen, Carlsbad, CA, USA). RNA was quantified using NanoDrop 2000 (Thermo Scientific, MA, USA), and 1 μg was run on a 1% agarose gel to check for RNA integrity. Complementary DNA (cDNA) was synthesized using iScriptTM Advanced cDNA Synthesis Kit (Bio-Rad Laboratories, CA, USA), according to manufacturer’s instructions. Real Time-quantitative PCR (RT-qPCR) was performed using the SsoFast EvaGreen Supermix or the SsoFast EvaGreen low ROX (Bio-Rad Laboratories, CA, USA) and the primer pairs described in **Supplementary Table 1**. Expression of target and housekeeping genes was analyzed on CFXTM Manager (Bio-Rad Laboratories) using the “Gene Study” function or on the 7500 Fast Real-Time PCR System using the 7500-software version 2.3 (Applied Biosystems by Life Technologies) and exported to Microsoft Excel for further calculations. *18S rRNA*, *Gapdh* and *Hprt* were used as housekeeping genes. The expression of *Gapdh* did not comply with the requirements to be used as housekeeping gene and was excluded from the analysis. Target genes expression presented in graphs are relative to the expression of the housekeeping genes *18S rRNA* and *Hprt*.

### Statistical Analysis

Results were expressed as mean or mean + SD, unless otherwise said. Prism 8.4.3 (GraphPad, CA, USA) was used for all the statistical analysis. Variables’ normality was accessed by Kolmogorov-Smirnov test. Tests were used as indicated in figure legends: two-tailed unpaired *t*-test, Mann-Whitney test, two-tailed ratio paired *t*-test, ordinary one-way ANOVA followed by Dunnett’s multiple comparisons test, or 2-way ANOVA followed by Tukey’s multiple comparisons test. Differences between groups were considered statistically significant for *p-*values <0.05.

## Results

### Increased Proportion of LSK Cells in the BM of Mice Infected With *M. avium* Strain 25291 Requires IFNγ but Not iNOS

The differentiation of new T cells is dependent on the continuous seeding of BM T cell precursors in the thymus. We hypothesized that *M. avium* infection causes alterations on T cell precursors which contribute to infection-induced thymic atrophy. To test this hypothesis, we analyzed the BM from WT mice infected with *M. avium* strain 25291, which induces premature thymic atrophy, or with *M. avium* strain 2447, which does not cause premature thymic atrophy. Additionally, BM from mice lacking IFNγ (IFNγKO), iNOS (iNOSKO) or the signaling of IFNγ in Mϕ (MIIG), crucial factors that contribute to *M. avium* strain 25291 infection-induced thymic atrophy (10), were also analyzed after infection.

**Figure 1:**
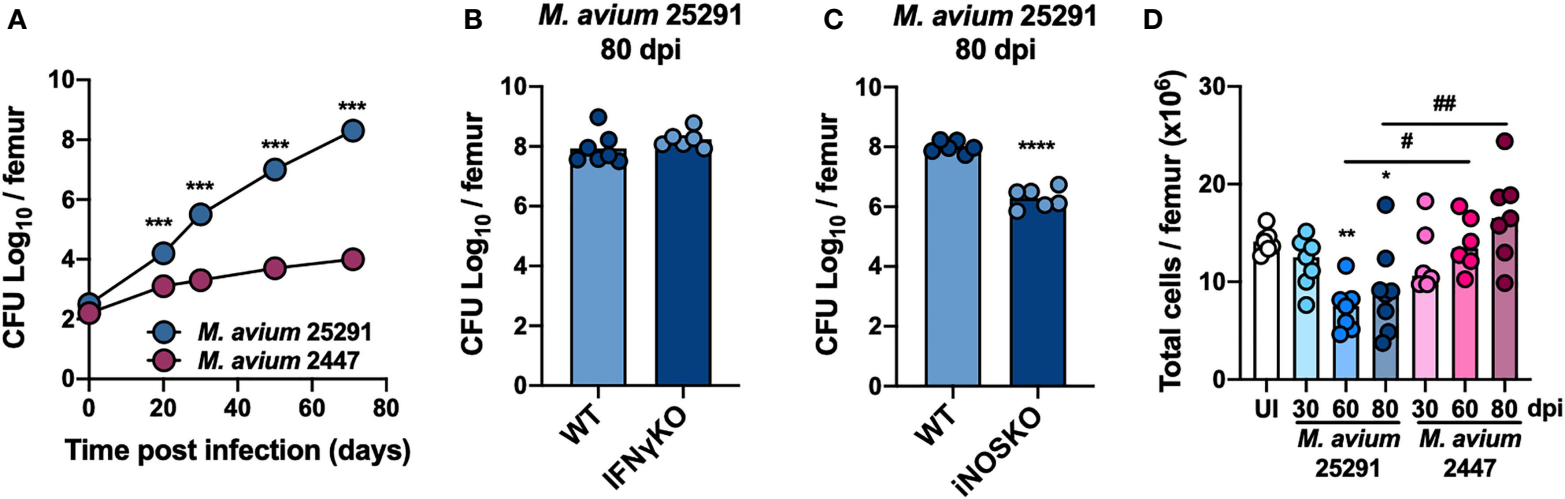
Bacteria load in the BM is higher after infection with *M. avium* strain 25291 than with strain 2447. **(A)** Representative kinetics of *M. avium* infection in the BM of WT mice infected with strains 25291 (blue) or 2447 (pink). **(B, C)** Bacterial load in the BM from WT, IFNγKO and iNOSKO mice infected with *M. avium* strain 25291 for 80 days. At each time-point of infection, groups were compared by two-tailed unpaired *t*-test and were marked as: ***p <0.001; ****p <0.0001. **(D)** Total number of BM cells per femur of uninfected WT mice (white), or after infection with *M. avium* strain 25291 (blue) or strain 2447 (pink) at 30, 60 and 80 dpi. The groups of infected mice, at the several time-points, were compared with the uninfected mice by ordinary one-way ANOVA followed by Dunnett’s multiple comparisons test and were marked as: *p <0.05; **p <0.01; comparisons between infected groups were performed by 2-way ANOVA followed by Tukey’s multiple comparisons test, and marked as: ^#^ *p <*0.05, ^##^ *p <*0.01. Data represent the mean ± SD **(A)** or mean **(B–D)** from 5 to 8 mice per group, from one of two independent experiments. UI stands for uninfected. dpi stands for days post infection.

We observed that the bacterial load in the BM from mice infected with strain 25291 is higher compared to that of mice infected with strain 2447, reaching a 5-log difference at 70 days post-infection (dpi; **Figure 1A**). At 80 dpi with *M. avium* strain 25291, mice lacking IFNγ have similarly high bacterial load in their BM compared to WT mice (**Figure 1B**), and iNOSKO mice have a 2-log lower bacterial load than WT mice (**Figure 1C**), in agreement with the bacterial load in the liver, spleen, lung and thymus (10, 11). Infection of WT mice with strain 25291 leads to decreased number of total BM cells (**Figure 1D**), as previously described (46). On the other hand, the total number of BM cells from WT mice infected with strain 2447 does not differ from those of uninfected animals through 80 dpi (**Figure 1D**).

**Figure 2:**
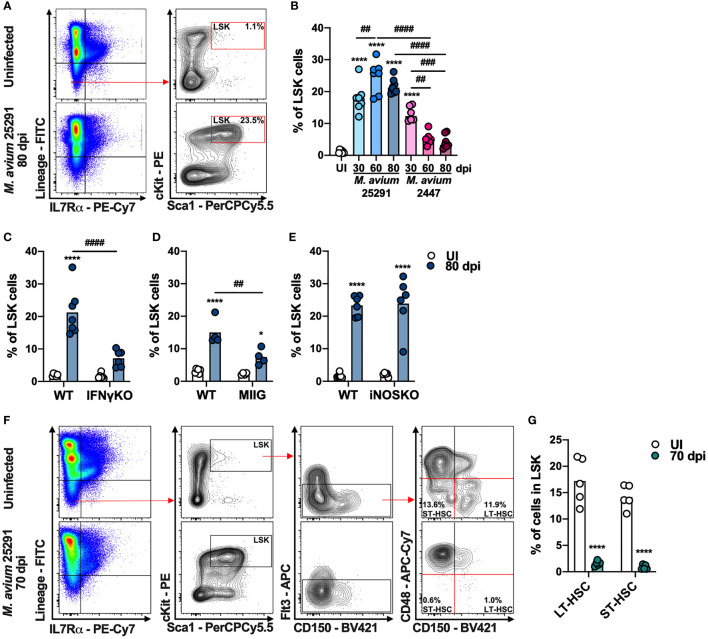
Mice infected with *M. avium* strain 25291 have higher percentage of LSK cells in an IFNγ-dependent and iNOS-independent manner. **(A)** Schematic representation of the gating used to identify LSK cell population in the BM from uninfected WT mice (top), or infected for 80 days with *M. avium* 25291 (bottom). Total BM cells were previously selected eliminating doublets and debris. **(B)** Percentage of LSK cells from uninfected WT mice (white) or after infection with *M. avium* strain 25291 (blue) or strain 2447 (pink) at 30, 60 and 80 dpi. Comparisons between infected and uninfected mice were performed using ordinary one-way ANOVA followed by Dunnett’s multiple comparisons test, and marked as: ****p < 0.0001; comparisons between all the infected groups were performed using a 2-way ANOVA followed by Tukey’s multiple comparisons test, and marked as: ^##^ *p < *0.01, ^###^ *p <*0.001, ^####^ *p < *0.0001. **(C–E)** Percentage of LSK cells from uninfected (white) or infected for 80 days with *M. avium* 25291 (blue) of WT, IFNγKO, MIIG or iNOSKO mice. Groups were compared using 2-way ANOVA followed by Tukey’s multiple comparisons test; statistical differences between uninfected and infected mice were marked as *p < 0.05 and ****p < 0.0001; and between infected groups as ^##^ *p < *0.01, ^####^ *p < *0.0001. **(F)** Schematic representation of the gating used to identify LT-HSC and ST-HSC cell populations in the BM from uninfected WT mice (top), or infected for 70 days with *M. avium* 25291 (bottom) **(G)** Percentage of LT-HSC and ST-HSC cells in WT uninfected (white) or after 70 days of infection with *M. avium* 25291 (teal). Comparisons between infected and uninfected were performed by two-tailed unpaired *t*-test and marked as ****p < 0.0001. Bars represent the mean from 4 to 8 mice per group from one of two independent experiments. UI stands for uninfected. dpi stands for days post infection.

To examine T cell precursors in the BM during infection with *M. avium* strain 25291, we analyzed the different BM cell populations by flow cytometry. Hematopoietic stem cells (HSC), which are part of the LSK (Lineage^-^ Sca1^+^ cKit^+^; see gating strategy, **Figure 2A**) population, possess the capacity for self-renewal and can give rise to all mature blood cell types (47). In comparison to uninfected mice, animals infected with *M. avium* strain 2447 have a transient increase in LSK cells percentage at 30 dpi, which return to normal levels over time. In contrast, in mice infected with the high virulence strain 25291, there is a sustained increase in the percentage of LSK cells throughout the time course evaluated (**Figure 2B**). Consistent with the described induction of LSK cell expansion mediated by IFNγ (39), there is no alteration on LSK cells percentage in IFNγKO mice infected with *M. avium* strain 25291 up to 80 dpi (**Figure 2C**). To understand if the increase in the percentage of LSK population is due to a direct effect of IFNγ, or another mechanism dependent on Mϕ activation, MIIG mice were infected. At 80 dpi, MIIG mice show increased LSK cells percentage when compared to uninfected mice. However, the percentage of LSK cells is lower on MIIG in comparison to WT mice (**Figure 2D**). As for iNOSKO mice, we show that the percentage of LSK cells increase upon infection with strain 25291 (80 dpi), similarly to WT mice (**Figure 2E**). Together, these results show that the increase on LSK cells percentage during infection by *M. avium* strain 25291 is partially dependent on IFNγ-induced activation of Mϕ but not on iNOS production. Finally, infection by *M. avium* strain 25291 leads to a dramatic decrease in the frequency of long term (LT)-HSC and short term (ST)-HSC (**Figures 2F, G**).

### Increased LMPP and Decreased CLP in the BM After Infection With *M. avium* 25291 Is Independent on IFNγ and iNOS

**Figure 3:**
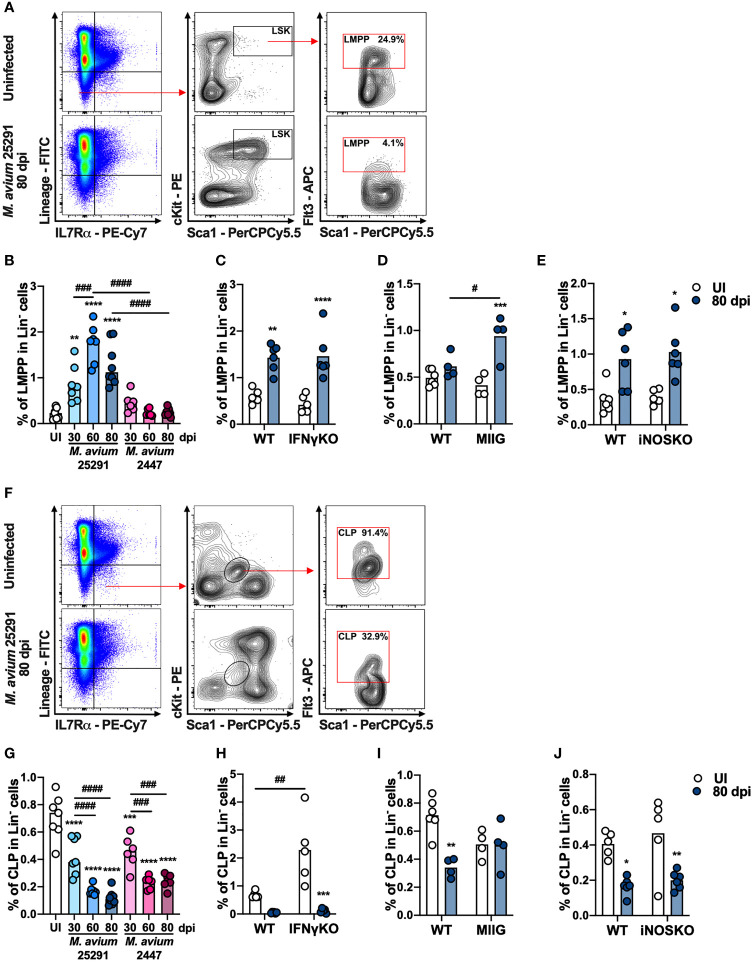
The increase on the percentage of LMPP and the decrease on the percentage of CLP in mice infected with *M. avium* 25291 is independent on IFNγ and iNOS. **(A)** Schematic representation of the gating used to select LMPP population in the BM from uninfected WT mice (top), or infected for 80 days with *M. avium* 25291 (bottom). Total BM cells were previously selected eliminating doublets and debris. **(B)** Percentage of LMPP in the lineage negative population from uninfected (white) WT mice or infected with *M. avium* strain 25291 (blue) or strain 2447 (pink) at 30, 60 and 80 dpi. **(C–E)** Percentage of LMPP in the lineage negative population from uninfected (white) or infected for 80 days with *M. avium* 25291 (blue), WT, IFNγKO, MIIG or iNOSKO mice. **(F)** Schematic representation of the gating used to select CLP population in the BM from uninfected WT mice (top), or infected for 80 days with *M. avium* 25291 (bottom). Total BM cells were selected eliminating doublets and debris. **(G)** Percentage of CLP in the lineage negative population from uninfected WT mice (white), or infected with *M. avium* strain 25291 (blue) or strain 2447 (pink) at 30, 60 and 80 dpi. **(H–J)** Percentage of CLP in the lineage negative population from uninfected (white) or infected for 80 days with *M. avium* 25291 (blue), WT, IFNγKO, MIIG or iNOSKO mice. In all graphs, bars represent the mean from 4 to 8 mice per group, from one of two independent experiments. In B and G comparisons between infected and uninfected mice were evaluated by ordinary one-way ANOVA followed by Dunnett’s multiple comparisons test and marked as: **p < 0.01, ***p <0.001, ****p < 0.0001; comparisons between infected groups were evaluated by 2-way ANOVA followed by Tukey’s multiple comparisons test and marked as: ^###^ *p < *0.001, ^####^ *p < *0.0001. In **(C–E, H–J)**, comparisons were evaluated by 2-way ANOVA followed by Tukey’s multiple comparisons test and marked as: *p < 0.05, **p < 0.01, *** *p < *0.001, ****p < 0.0001 for comparisons between uninfected and infected, and as: ^#^ *p* < 0.05, ^##^ *p* < 0.01, ^###^ *p < *0.001, ^####^ *p < *0.0001 for comparisons between infected groups. UI stands for uninfected. dpi stands for days post infection.

There are two BM cell populations described as able to differentiate into T cells *in vitro* and *in vivo* (48): (i) the lymphoid-primed multipotent progenitors (LMPP), which is a subpopulation of LSK cells that express Flt3 (Lin^-^ IL-7Rα^-^ Sca1^+^ cKit^+^ Flt3^+^); and (ii) the common lymphoid progenitors (CLP), which are Lin^-^ IL-7Rα^+^ Sca1^int^ cKit^int^ Flt3^+^. Compared to uninfected mice, the percentage of LMPP (see gating strategy, **Figure 3A**) is increased after infection with *M. avium* strain 25291, but not with strain 2447 (**Figure 3B**). We also observed an increase in the number of LMPP with both *M. avium* strain 25291 and 2447 that is sustained only for strain 25291 (**Supplementary Figure 1A**). The percentage and number of LMPP is not different in *M. avium* strain 25291 infected IFNγKO (**Figure 3C** and **Supplementary Figure 1B**), MIIG (**Figure 3D** and **Supplementary Figure 1C**) and iNOSKO (**Figure 3E** and **Supplementary Figure 1D**) mice comparing with WT infected mice. These results show that the increased proportion of LMPP does not require IFNγ nor iNOS expression and thus is not sufficient to cause infection-induced thymic atrophy.

We observed an obvious decrease in both percentage and number of CLP (see gating strategy, **Figure 3F**) in WT mice infected with *M. avium* strain 25291, a change that is also observed in mice infected with the low virulence strain 2447 (**Figure 3G** and **Supplementary Figure 1E**). We also observed a reduction in the percentage of CLP in *M. avium* 25291 infected IFNγKO mice (**Figure 3H**). But the decrease in the percentage of CLP upon infection with strain 25291 is dependent on the activation of Mϕ by IFNγ, since MIIG mice have the same percentage of CLP as uninfected mice (**Figure 3I**). iNOSKO mice infected with *M. avium* strain 25291 have a reduction in the percentage of CLP similar to WT infected mice (**Figure 3J**). The number of CLP is reduced in IFNγKO, MIIG and iNOSKO mice, as observed in WT mice, at 80 dpi (**Supplementary Figures 1F–H**). These results show that IFNγ and the production of NO, are not required for the CLP alterations upon *M. avium* strain 25291 infection.

To assess other molecules that might be involved in the observed alterations in BM cell populations, we analyzed the expression of genes previously described to affect the BM in various infection/inflammation models. We observed that interleukine-6(*Il6*) expression is increased in WT mice infected with *M. avium* strain 25291 compared to uninfected mice but not in infected iNOSKO mice (**Supplementary Figure 2A**). No significant differences were observed after infection in the expression of glucocorticoid receptor (*Gr*) and tumor necrosis factor (*Tnf*) in the BM (**Supplementary Figures 2B, C**).

### BM Cells From WT Mice Infected With *M. avium* 25291 Poorly Reconstitute Thymi From RAGKO Mice

**Figure 4:**
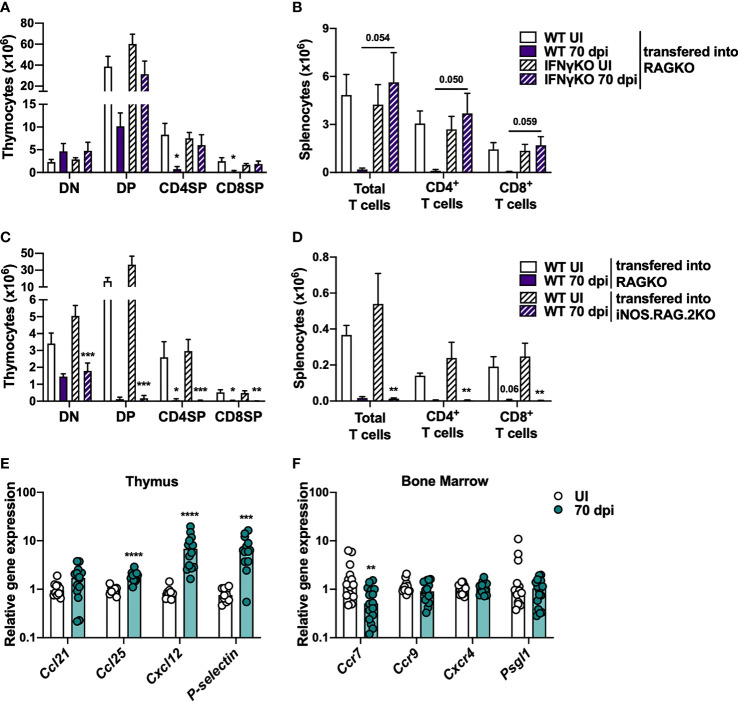
Overall decreased ability to reconstitute thymi from RAGKO mice by BM cells from *M. avium* strain 25291 infected mice is IFNγ-dependent. **(A, B)** RAGKO mice were reconstituted with lineage negative BM cells from uninfected (white) or 70 days *M. avium* 25291 infected (purple) WT (solid) or IFNγKO mice (dashed). **(A)** Number of the four main thymocyte populations (DN, DP, CD4SP and CD8SP). **(B)** Number of splenic total T cells, and of CD4^+^ and CD8^+^ T cells. **(C, D)** RAGKO (solid) or iNOS.RAG.2KO (dashed) mice were reconstituted with lineage negative BM cells from uninfected (white) or 70 days *M. avium* 25291 infected (purple) WT mice. **(C)** Number of cells from the four main thymocyte populations. **(D)** Number of splenic T cells and its subpopulations. RNA expression levels in WT mice uninfected (white) or infected with *M. avium* strain 25291 for 70 days (teal). **(E)** Expression in the thymus of the chemokines *Ccl21*, *Ccl25* and *Cxcl12*, and of *P-selectin*. **(F)** Expression in the BM of the chemokines receptors *Ccr7*, *Ccr9* and *Cxcr4*, and of *Psgl1*. In **(A–D)** bars represent the mean + SEM from 3 to 6 mice per group from one of two independent experiments. Comparisons were performed using 2-way ANOVA followed by Tukey’s multiple comparisons test, and marked between infected and uninfected mice as: *p < 0.05, **p < 0.01, ***p < 0.001. For *p*-values between [0.05; 0.10], their values are represented in the graphs. In E and F bars represent the median from 10 to 16 mice per group from two pooled independent experiments. Comparisons between infected and uninfected were performed by two-tailed unpaired *t*-test or Mann-Whitney test, according to normality, and marked as **p < 0.01, ***p < 0.001, ****p < 0.0001. UI stands for uninfected. dpi stands for days post infection. DN stands for double negative. DP stands for double positive. SP stands for single positive.

Since the observed reduction on the percentage and number of CLP is independent of IFNγ and iNOS, this alteration was excluded as the mechanism responsible *per se* for *M. avium*-induced thymic atrophy; still it might synergize with other mechanisms yet to be identified. To investigate if alterations in BM precursors contribute to *M. avium* infection-induced thymic atrophy, we transferred lineage negative BM cells from uninfected or 70 days infected WT or IFNγKO mice, into RAGKO recipients. Recipient mice were euthanized 4 weeks later. Mice receiving BM cells from WT mice infected with *M. avium* 25291 present an overall weaker thymic reconstitution than mice receiving BM cells from uninfected mice or from IFNγKO infected and uninfected mice (**Figure 4A**). The observation that BM from infected IFNγKO mice has the same ability to reconstitute the thymus as WT uninfected reveals that the reduced ability to reconstitute the thymus of infected WT BM cells is not dependent on the altered percentage/number of CLP and LMPP. In agreement with the data from thymic reconstitutions, a lower number of total splenocytes, including total and both CD4^+^ and CD8^+^ T cells, were recovered from mice receiving BM from infected WT mice, compared to mice receiving BM cells from uninfected WT or IFNγKO infected mice (**Figure 4B**).

To test if the reconstitution of BM precursor cells from WT infected mice is inhibited by NO production in the recipient thymus, lineage negative BM cells from WT mice, uninfected or *M. avium* 25291 infected (70 dpi) were transferred to iNOS.RAG.2KO recipients. Irrespective of donor BM cells being from infected or uninfected mice, no differences between RAGKO and iNOS.RAG.2KO recipient mice were observed when comparing the reconstitution of the thymus (**Figure 4C**), and the T cell pool in the spleen (**Figure 4D**).

The recruitment and entry of BM T cell precursors to the thymus is mediated by chemokines expressed by thymic epithelial cells (TEC), such as CCL21, CCL25 and CXCL12, as well as P-selectin. These molecules are recognized by their receptors in precursor cells, such as CCR7, CCR9, CXCR4 and PSGL1. The analysis of the expression of these molecules revealed increased expression of *Ccl25*, *Cxcl12 and P-selectin* in the thymus (**Figure 4E**), and decreased expression of *Ccr7* in BM cells (**Figure 4F**) upon *M. avium* strain 25291 infection.

Overall, these results show that BM T cell precursors from mice infected for 70 days with *M. avium* 25291 have a lower ability to reconstitute mice lacking T cells in a process dependent on the expression of IFNγ on BM cells but independent of NO production by the recipient thymus. The data presented here support the hypothesis that infection-induced BM cells alterations play a relevant part on thymic atrophy caused by *M. avium* infection.

### IFNγ and iNOS Expression Is Associated With a More Inflammatory Profile in the Thymus After Infection

**Figure 5:**
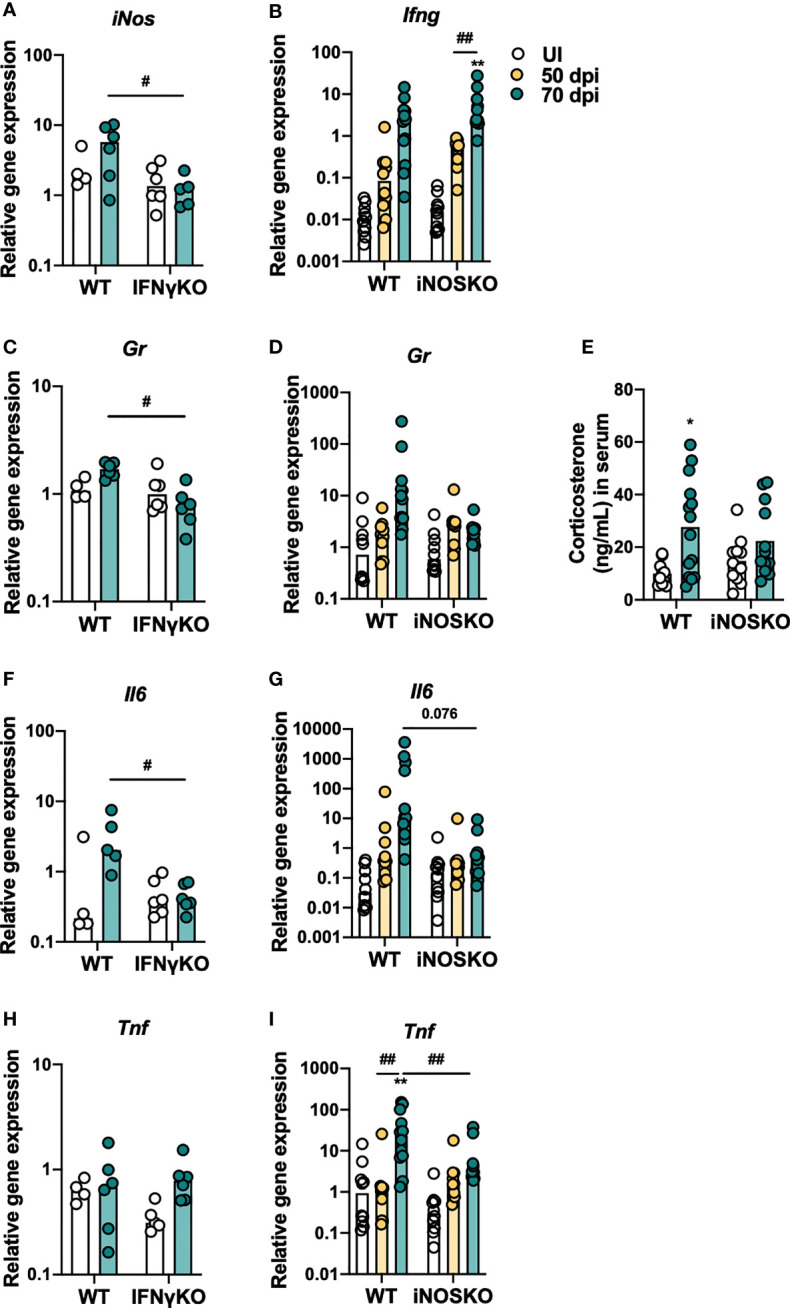
*M. avium* strain 25291 infected IFNγKO and iNOSKO mice present a milder inflammatory profile in the thymus than WT infected mice. RNA expression levels in the thymus of WT, IFNγKO and iNOSKO mice uninfected (white) or infected with *M. avium* strain 25291 for 50 (yellow) or 70 (teal) days. **(A)** *iNos*; **(B)** *Ifng*; **(C, D)** *Gr*; **(F, G)** *Il6;* and **(H, I)** *Tnf.* **(E)** Basal corticosterone levels in the serum of WT and iNOSKO mice uninfected (white) or infected with *M. avium* strain 25291 for 70 days (teal). Bars represent the median for all graphs except for **(E)** where mean is represented; for **(A, C, F, H)** from 4 to 6 mice per group from one experiment; for **(B, D, E, G, I)** from 10 to 12 mice per group from two pooled independent experiments. Statistically significant differences were accessed by 2-way ANOVA followed by Tukey’s multiple comparisons test and marked between uninfected and infected groups as: *p < 0.05, **p < 0.01; and between infected groups as: ^#^ *p < *0.05, ^##^ *p < *0.01. For *p*-values between [0.05; 0.10], their values are represented in the graphs. UI stands for uninfected. dpi stands for days post infection.

The observation that IFNγKO and iNOSKO mice have the same alterations on the percentage/number of BM T cell precursors as WT mice after infection, but no premature thymic atrophy (10), suggests that these alterations *per se* are insufficient to cause premature thymic atrophy. Therefore, we hypothesized that IFNγ and NO production have a direct impact in the thymus that synergizes with alterations in the BM precursor cells. To investigate this hypothesis, the expression of inflammatory molecules in the thymus of WT, IFNγKO and iNOSKO mice was evaluated after infection with *M. avium* strain 25291. We observed lower expression of *iNos* in the thymus of infected IFNγKO mice compared to infected WT mice (**Figure 5A**), while *Ifng* expression is increased upon infection, independently of iNOS expression (**Figure 5B**). In respect to *Gr* expression, no clear differences were observed upon infection with *M. avium* strain 25291 (**Figures 5C, D**), although WT, but not iNOSKO infected mice, show higher serum levels of corticosterone (**Figure 5E**). Infection induces the expression of *Il6* in WT mice but not in IFNγKO and iNOSKO mice (**Figures 5F, G**). We also observed a tendency for increased *Tnf* expression in the thymus of WT, not present in iNOSKO mice (**Figures 5H, I**), despite high variability for this particular gene within groups and between experiments.

### Thymic Stroma From *M. avium* 25291 Infected Mice Is Unable to Support Optimal T Cell Differentiation

**Figure 6:**
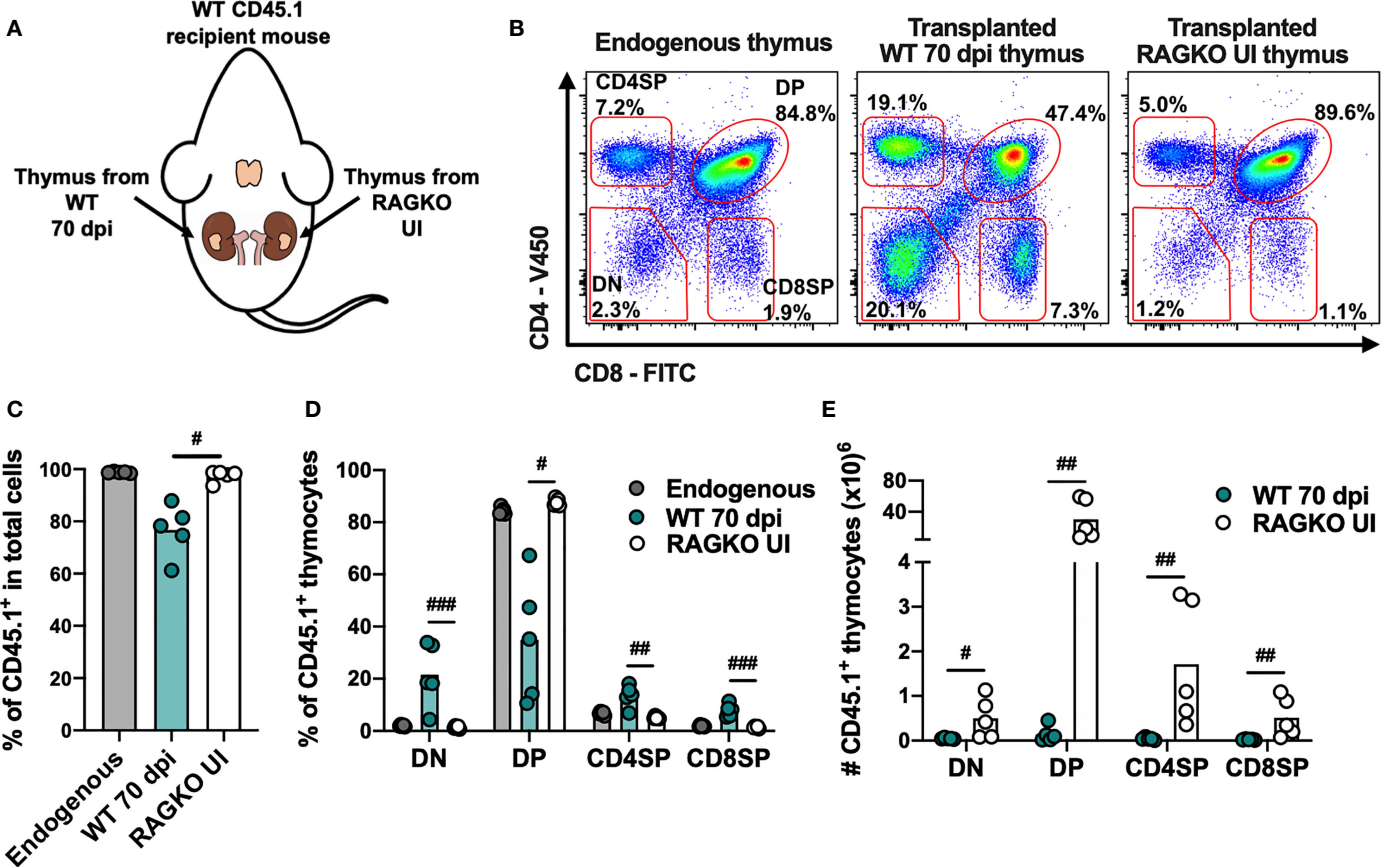
Thymic stroma from WT mice infected with *M. avium* 25291 does not properly support T cell differentiation. **(A)** Experimental schematic representation of thymi transplant from *M. avium* infected WT mice (70 dpi) and uninfected RAGKO mice (both CD45.2) under the kidney capsule of WT CD45.1 recipient mice. Mice were euthanized 4 weeks after transplant. **(B)** Representative plots of the CD45.1^+^ four main thymocyte populations (DN, DP, CD4SP and CD8SP) from WT CD45.1 recipient mice endogenous thymi, and from transplanted WT infected and RAGKO uninfected thymi. **(C)** Percentage of cells recruited from the recipient mouse (CD45.1^+^), and **(D)** percentage and **(E)** number of cells from the four main thymocyte populations from endogenous thymi of WT CD45.1 recipient mice (grey), or from transplanted thymi from 70 days *M. avium* 25291 infected WT mice (teal) or uninfected RAGKO mice (white). Data represent the mean from five mice per group from one of two independent experiments. Comparisons between WT 70 dpi and uninfected RAGKO were performed by 2-tailed ratio paired t test and marked as: ^#^ *p < *0.05, ^##^ *p < *0.01, ^###^ *p < *0.001. UI stands for uninfected. dpi stands for days post infection. DN stands for double negative. DP stands for double positive. SP stands for single positive.

To determine if alterations in the thymic stroma (considered here as composed by TECs, antigen presenting cells and the overall milieu) contribute to infection-induced thymic atrophy, we investigated the ability of thymi from infected animals to support thymocyte differentiation when BM cells from uninfected mice are provided. Thus, atrophied thymi from infected WT mice (CD45.2) and from uninfected RAGKO mice (CD45.2), were transplanted under the kidney capsules of uninfected WT CD45.1 mice (i.e., one thymus on each kidney of the same recipient mouse; **Figure 6A**). Four weeks after transplant mice were euthanized and the four main thymocyte populations within CD45.1^+^ cells were analyzed in the transplanted thymi and in the endogenous thymi of recipient mice (**Figure 6B**). Transplanted thymi from infected WT mice show lower percentage of total thymocytes with origin in the recipient mice (CD45.1^+^), when compared with transplanted thymi from uninfected RAGKO mice. Still, precursor cells from recipient mice are recruited and replace most of the donor thymocytes in the transplanted thymi from infected WT mice, as the majority of cells recovered are CD45.1^+^(**Figure 6C**). The percentage of the thymocyte populations in the transplanted WT infected thymi are significantly distorted, while that in the transplanted RAGKO thymi are similar to the ones observed in the endogenous thymi (**Figure 6D**). The absolute number of each thymocyte population is higher in uninfected RAGKO thymi than in thymi from infected WT mice (**Figure 6E**). These results show that thymic stroma from WT mice infected with *M. avium* 25291 has an impaired ability to support thymocyte differentiation.

Upon 70 days of infection by *M. avium* strain 25291, a disruption of the thymic structure is observed, although there is variability between mice. While some infected WT mice maintain a compartmentalized thymic structure, with clear distinction between cortex (cTECs, stained mostly by K8) and medulla (mTECs, stained mostly by K5), others present a thymus without clear distinction of the two main compartments and densely stained for K5 and K8 (**Supplementary Figure 3**). However, even in infected WT mice that preserve a clear compartmentalization between cortex and medulla, there is a reduction of the cortex region, as observed in K8 and HE stains (**Supplementary Figure 3**).

### Increased Thymocyte Death in Infected Mice Is Independent of Caspase-3 Activation

**Figure 7:**
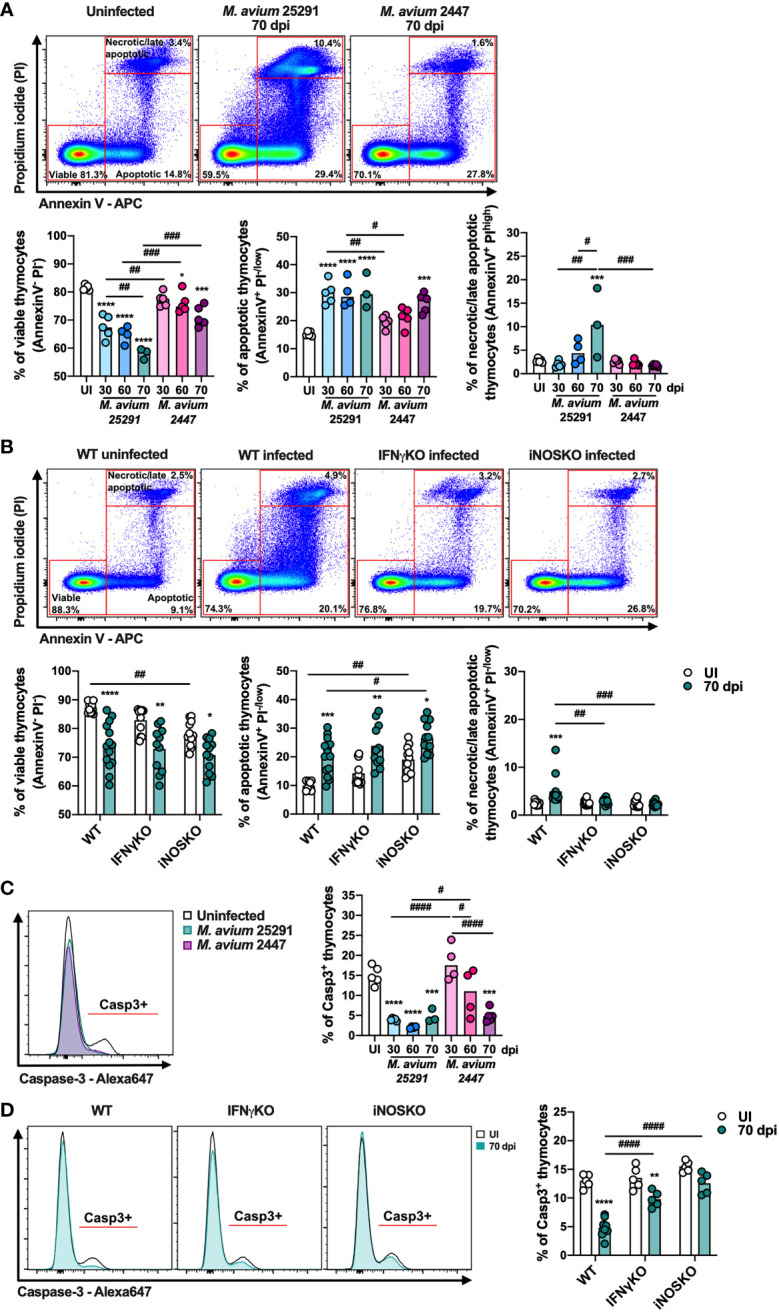
The percentage of viable cells and of cells positive for active caspase-3 decrease after infection. **(A)** Representation of the gating (top) and plotting of viable (left graph – AnnexinV^-^ PI^-^), apoptotic (center graph – AnnexinV^+^ PI^-/low^) and necrotic/late apoptotic (right graph – AnnexinV^+^ PI^high^) thymocytes from uninfected WT mice (white) or infected for 30, 60 or 70 days with *M. avium* 25291 (blue) or *M. avium* 2447 (pink). **(B)** Representation of the gating (top) and plotting of viable (left graph – AnnexinV^-^ PI^-^), apoptotic (center graph – AnnexinV^+^ PI^-/low^) and necrotic/late apoptotic (right graph – AnnexinV^+^ PI^high^) thymocytes from WT, IFNγKO or iNOSKO mice uninfected (white) or infected for 70 days with *M. avium* 25291 (teal). **(C)** Representative histogram and plotting of active caspase-3 positive thymocytes from uninfected WT mice (white) or infected for 30, 60 or 70 days with *M. avium* 25291 (blue) or *M. avium* 2447 (pink). **(D)** Representative histogram and plotting of caspase-3 positive thymocytes from WT, IFNγKO or iNOSKO mice uninfected (white) or infected for 70 days with *M. avium* 25291 (teal). For all the analysis, total thymocytes were previously selected eliminating doublets and debris. Bars represent the mean from: 3 to 5 mice per group from one experiment for **(A, C)**; 9 to 14 mice per group from two pooled independent experiments for **(B, D)**. In **(A, C)** comparisons between infected and uninfected mice were evaluated by ordinary one-way ANOVA followed by Dunnett’s multiple comparisons test and marked as: *p < 0.05, ***p < 0.001, ****p < 0.0001; and comparisons between infected groups were evaluated by 2-way ANOVA followed by Tukey’s multiple comparisons test and marked as: #*p < *0.05, ^##^ *p < *0.01, ^###^ *p < *0.001, ^####^ *p < *0.0001. In **(B, D)**, comparisons were performed by 2-way ANOVA followed by Tukey’s multiple comparisons test, and marked as *p < 0.05, **p <0.01, ***p < 0.001, ****p < 0.0001 for differences between uninfected and infected, and as #*p < *0.05, ^##^ *p <*0.01, ^###^ *p < *0.001, ^####^ *p < *0.0001 for differences between infected groups. UI stands for uninfected. Casp3 stands for active caspase-3. dpi stands for days post infection.

To further dissect the mechanisms that lead to infection-induced thymic atrophy we investigated thymocyte death by analyzing the incorporation of propidium iodide (PI) and binding of annexin V by thymocytes. Infection by both *M. avium* strains (25291 and 2447) leads to a reduction in the percentage of viable cells (Annexin V^-^ PI^-^) and an increase in the percentage of apoptotic cells (Annexin V^+^ PI^-/low^) (**Figure 7A**). However, these alterations occur earlier and are of a greater magnitude in mice infected with the high virulence strain 25291 compared to mice infected with the low virulence strain 2447 (**Figure 7A**). Increased percentage of thymocytes undergoing necrosis/late apoptosis (Annexin V^+^ PI^high^) is observed upon infection with *M. avium* strain 25291, but not with strain 2447 at 70 dpi (**Figure 7A**).

To understand if these alterations are dependent on IFNγ and/or NO production, we analyzed thymocyte cell death in thymi from IFNγKO and iNOSKO mice at 70 dpi. The reduction in the percentage of viable thymocytes, and the increase in the percentage of apoptotic thymocytes is similarly observed in IFNγKO and iNOSKO mice, indicating that these alterations in death do not require IFNγ or NO (**Figure 7B**). However, neither IFNγKO nor iNOSKO infected mice reveal an increased percentage of necrotic/late apoptotic thymocytes in comparison to the uninfected peers (**Figure 7B**). With respect to the four main thymocyte populations, DP, CD4 single positive (SP) and CD8SP are the ones most affected after infection, in an IFNγ and NO independent manner (**Supplementary Figure 4A-D**). In infected WT mice, DP thymocytes is the only population with increased percentage of necrosis/late apoptosis, a difference not present in IFNγKO or iNOSKO infected mice, as observed in total thymocytes (**Supplementary Figure 4B**).

Finally, we observed that infection with both *M. avium* strains leads to decreased percentage of active caspase-3 positive thymocytes. In mice infected with strain 25291, this decrease is evident as early as 30 dpi, and is sustained up to 70 dpi. In contrast, the reduction in the percentage of active caspase-3 positive thymocytes is only observed at 70 dpi in mice infected with strain 2447 (**Figure 7C**). Upon infection with strain 25291, the percentage of active caspase-3 positive thymocytes decreases in IFNγKO mice, though to a lower extent in comparison to WT mice (**Figure 7D**). These results show that the increased apoptosis occurring in thymocytes after infection is independent of caspase-3 activation. Additionally, the reduction in the percentage of active caspase-3 positive thymocytes is partially dependent of IFNγ and dependent of iNOS.

## Discussion

Infection by *M. avium* strain 25291 results in severe thymic atrophy, a process dependent on the synergy between GC and NO produced by IFNγ activated Mϕ (10). Here we investigate where and how these mediators cause *M. avium*-induced thymic atrophy

Following infection with *M. avium* strain 25291 we found an increase in the percentage of LSK cells in the BM which is dependent on IFNγ. Our results are coherent with previous reports describing LSK expansion during infection by *M. tuberculosis*, *M. avium* and Vaccinia virus (29, 30, 35, 39). To our knowledge, we report for the first time that the expansion of LSK cells during *M. avium* infection is at least partially dependent on Mϕ activation by IFNγ. The experiments leading to this conclusion were those using MIIG mice, whose CD68^+^ cells are defective in IFNγ signaling. Although an increase on the percentage of LSK cells can be observed at 80 dpi in MIIG mice, it does not reach the same magnitude of increase as that observed in infected WT mice. The expansion of LSK cells during *M. tuberculosis* infection has been associated with two cytokines produced by Mϕ, TNF and IL-6 (29). We also observed an increase in the mRNA expression of these two cytokines in the BM after *M. avium* strain 25291 infection, supporting the relevance of activated Mϕ in LSK expansion. We also show that the expansion of LSK cells is independent of iNOS, as iNOSKO infected mice present the same alteration in this BM population as WT infected mice. This shows that the expansion of LSK might be part of the mechanism leading to thymic atrophy but clearly not sufficient.

Within the LSK population, we observed a reduction in the percentage of LT-HSC and ST-HSC. HSC are very sensitive to the surrounding microenvironment and HSC alterations associated with infection and inflammation have been well described. These alterations include the reduction of the HSC pool and changes in their proliferation (30, 49–54). During chronic inflammation or chronic IFNγ signaling there is impaired self-renewal of HSC that culminates in the reduction of this population (49). We propose that this same mechanism leads to the reduction in HSC percentage observed upon infection with *M. avium* strain 25291.

Regarding the most direct T cell precursors in the BM, CLP and LMPP, we show that *M. avium* infection induces an increase in the percentage and number of LMPP, which is independent of IFNγ and iNOS. Kong *et al.*, using a sepsis model, described an increase in the number of LMPP associated with premature thymic atrophy (41), which is consistent with our results. In contrast, the percentage and number of CLP is reduced after *M. avium* infection with either strain 25291 or 2447, being clearly more evident for the more virulent strain 25291. This decrease of CLP is independent of IFNγ and iNOS. Reduction of CLP was also reported after *P. chabaudi* infection, although thymic atrophy was not assessed in this study (36). In contrast, infection with Vaccinia virus, known to cause thymic atrophy, leads to an increase of CLP in the BM (35). These results indicate that directionality of the changes in this BM population is pathogen specific and not always associated with infection-induced thymic atrophy.

BM resident Mϕ are essential for bone marrow niche homeostasis. Mϕ are in close contact with HSC and contribute for the maintenance of quiescence, self-renewal and proliferation of these cells (55, 56). BM resident Mϕ are also crucial for erythropoiesis (57), and it has been shown that infection with *M. avium* strain 25291 induces alterations in the production of erythrocytes and an accelerated removal of those cells from the circulation, resulting in anemia (46). Not only IFNγ impact the BM, other cytokines that are produced by Mϕ, such as IL-6, TNF and type I IFN, have been also associated with disturbances in BM precursors after infection and/or inflammation (29, 34, 40). In fact, both *Il6* and *Tnf* mRNA expression is increased in the BM of *M. avium* strain 25291 infected mice. It has been described that the overproduction of IL-6 after infection with Group B *Streptococcus* is associated with iNOS expression (58). We also show that up-regulation of *Il6* expression in the BM after infection with *M. avium* is dependent on iNOS. The activation of Mϕ might therefore play a role in infection-induced alterations in hematopoiesis, as is suggested by the results presented here.

Unlike BM cells from uninfected WT or IFNγKO infected mice, BM cells from *M. avium* strain 25291 infected WT mice have an overall impaired ability to reconstitute the thymus and the periphery of RAGKO mice. One could associate the reduced ability of BM cells from infected WT mice to reconstitute the thymus from RAGKO mice with the reduced number of CLP after infection. However, infected IFNγKO mice show the same reduction in CLP, and the BM cells from these mice reconstitute the thymus from RAGKO mice similarly to uninfected mice. These results show that during infection by *M. avium* 25291, changes in the overall ability of BM cells to progress into T cell differentiation contribute to premature thymic atrophy. To our knowledge, only two reports associate BM defects with premature thymic atrophy. One of them shows that sepsis-induced thymic atrophy is associated with a dramatic decrease in early thymic precursors (ETP) as a consequence of impaired migration of progenitors from the BM to the thymus, and the inability of BM progenitors to commit to the lymphoid lineage (41). A possibility is that infection might impair the migration and entry of BM T cell precursors into the thymus. Although we showed before a reduction on the number of ETPs in the thymus of infected mice (10), we saw no obvious differences on the expression of chemokine receptors responsible for the migration of BM T cell precursors, *Ccr9* and *Cxcr4*, nor on *Psgl1* that is needed for the entry of the precursors in the thymus, in BM cells from infected WT mice. Only the expression of the receptor *Ccr7* is decreased after infection, although no alterations were observed on the expression in the thymus of its chemokine ligand *Ccl21*. This is in contrast with a previous report on sepsis-induced thymic atrophy, in which the authors show a down-regulation of the expression of CCR7, CCR9 and PSGL1 (41).

The severe alterations in the BM during *M. avium* strain 25291 infection do not appear to be the sole cause of thymic atrophy. T cell differentiation only occurs in the thymic microenvironment, which is supported by TECs. By performing thymic transplants, we show that the stroma of *M. avium*-atrophied thymi are impaired in their capacity to support the differentiation of new T cells when provided with BM precursors from uninfected WT mice, which are able to give rise to T cells in thymic stroma of RAGKO mice. In a model of thymic atrophy during pregnancy, thymic stromal cells were shown to have limited capacity to produce chemokines essential for T cell precursors homing to the thymus, including CCL25, CXCL12, CCL21 and CCL19 (59). The limited ability to recruit BM T cell precursors to the thymus could be a mechanism consistent with our previous observation that a reduction on the most immature thymocytes (ETPs; T cell precursor cells that just entered the thymus) occurs after infection with *M. avium* strain 25291 (10). However, we show that the expression in the thymus of *Ccl25* and *Cxcl12* is increased after infection with *M. avium* strain 25291. *P-selectin*, that is essential for the rolling and entry of T cell precursors in the thymus, is also increased in thymi from infected WT mice. These data led us to propose that there are limitations in the thymic stroma to retain T cell precursors and/or support T cell differentiation itself. Long-term infection with the high virulence strain 25291 leads to alteration in the proportion of the two main thymic regions, namely a reduction in the cortex and a proportional increase in the medulla, that might simply be associated with lack of thymocytes. In some mice, but not all, thymi from 70 dpi reveal a lack of clear distinction between cortex and medulla and an overall increased expression of K5 and K8. This type of disorganized thymic structure has been previously observed during HIV (60–62), MuLV (63), *Leishmania infantum* (64) and *P. berghei* (65) infections.

It is possible that infection with *M. avium* strain 25291 impairs thymocyte differentiation itself, either by blocking a certain differentiation stage or by inducing thymocyte cell death. We observed increased expression of *Il6* in the thymus of infected mice, which is in accordance with a study showing that over-expression of IL-6 inhibits the differentiation of double negative (DN) thymocytes in a model of *T. cruzi* infection-induced thymic atrophy. Increased GC, IFNγ, TNF and NO levels have been associated with augmented thymocyte apoptosis during infection and other conditions (25–28, 66–68). We found a reduction in the percentage of viable (Annexin V^-^ PI^-^) thymocytes after infection, independent of IFNγ and iNOS. During T cell differentiation, all the non-positively selected thymocytes die by programmed cell death, which represents around 90% of the thymocytes. For this reason, the thymus is a specialized organ for dead cell clearance (69). This makes it challenging to study cell death in the thymus since dead cells are difficult to be tracked. This rapid clearance could explain why only a slight decrease in the percentage of viable thymocytes is observed after infection. Still, an increase in thymocyte death is consistent with most infection-induced thymic atrophy models, in which increased thymocyte apoptosis is suggested to be the main mechanism (19, 70–77). We detect an increase in *Tnf* mRNA expression in the thymus after infection with *M. avium* strain 25291, which was associated with thymocyte apoptosis in other models (26, 66, 67). However, the increase in thymocyte apoptosis was also observed in WT mice infected with *M. avium* strain 2447, and in IFNγKO and iNOSKO mice infected with strain 25291, all conditions without infection-induced thymic atrophy (10). This lack of correlation implies that while cell apoptosis may contribute to *M. avium*-induced thymic atrophy, it is not the sole mechanism. Additionally, we observed a decrease in the percentage of thymocytes positive for active caspase-3. Thus, while thymocytes are dying by apoptosis, apoptosis may be mediated by a mechanism other than activation of caspase-3. However, as caspase-3 is fundamental for T cell differentiation in the thymus (78), the reduction in the percentage of cells expressing the active form of this enzyme suggests that infection is affecting other unexplored parameters of thymocyte differentiation, in an iNOS dependent manner. Finally, WT infected mice have an increase in the percentage of necrotic/late apoptotic thymocytes, which is not observed in IFNγKO and iNOSKO infected mice. As this increase is only detected at late time points (70 dpi), and thymic atrophy is already evident earlier (10), this implies that the appearance of necrotic/late apoptotic thymocytes is not the driver of *M. avium*-induced thymic atrophy.

In conclusion, we show that the mechanism of *M. avium*-induced premature thymic atrophy results from the association of several factors that seems to be cumulative: (1) alterations of the T cell precursors in the BM; (2) impaired ability of BM precursors to commit to thymocyte differentiation once within the thymus; (3) reduced capacity of thymic stromal cells to sustain T cell differentiation; and (4) IFNγ- and iNOS-independent thymocyte apoptosis. Together, our data shows that infection with *M. avium* strain 25291 induces IFNγ production that alters BM T cell precursors but that other alterations, independent of IFNγ and iNOS, are also required. This is probably associated with the parallel production of IL-6, TNF and/or other pro-inflammatory cytokines. T cell precursors that reach the thymus encounter a harsh microenvironment affected by prolonged inflammation, which impairs their ability to differentiate, and/or leads to death, and culminates in premature thymic atrophy.

## Data Availability Statement

The raw data supporting the conclusions of this article will be made available by the authors, without undue reservation.

## Ethics Statement

The animal study was reviewed and approved by in accordance with the recommendations of the European Convention for the Protection of Vertebrate Animals Used for Experimental and Other Scientific Purposes (ETS 123) and 86/609/EEC Directive and Portuguese rules (DL 129/92). The animal facility and people directly involved in animal experiments were certified by the Portuguese regulatory entity - Direção Geral de Alimentação e Veterinária (DGAV); the animal experimental protocols were approved by DGAV (# 015584), or by the Institutional Animal Care and Use Committee at the University of Massachusetts Medical School (Animal Welfare A3306-01), using the recommendations from the Guide for the Care and Use of Laboratory Animals of the National Institutes of Health and the Office of Laboratory Animal Welfare.

## Author Contributions

PB-S, MC-N, and RA conceptualized, designed, and supervised the study. PB-S performed the experiments and processed the samples, analyzed the data, prepared the figures, performed the statistical analysis, and drafted the first version of the manuscript. RM-M, CN, SR, CS-M, MB, GA, and DS collected and processed samples. MC-N, RA, and SB assembled the funding for experiments. All authors contributed to the article and approved the submitted version.

## Funding

This work has been funded by National funds, through the Foundation for Science and Technology (FCT) - project UIDB/50026/2020 and UIDP/50026/2020; by ICVS Scientific Microscopy Platform, member of the national infrastructure PPBI - Portuguese Platform of Bioimaging (PPBI-POCI-01-0145-FEDER-022122); and by a doctoral fellowship to PB-S (SFRH/BD/73544/2010). Experiments performed at University of Massachusetts Medical School were funded through a grant from the National Institutes of Health, R01 AI106725.

## Conflict of Interest

The authors declare that the research was conducted in the absence of any commercial or financial relationships that could be construed as a potential conflict of interest.

## Publisher’s Note

All claims expressed in this article are solely those of the authors and do not necessarily represent those of their affiliated organizations, or those of the publisher, the editors and the reviewers. Any product that may be evaluated in this article, or claim that may be made by its manufacturer, is not guaranteed or endorsed by the publisher.
