# Supplementary material for: IFNγ and iNOS-Mediated Alterations in the Bone Marrow and Thymus and Its Impact on Mycobacterium avium-Induced Thymic Atrophy
Source: Front Immunol. 2021 Dec 20;12:696415. doi: 10.3389/fimmu.2021.696415 (PMC8721011; doi:10.3389/fimmu.2021.696415)
Supplement: Supplementary file 1 [file DataSheet_1.pdf]

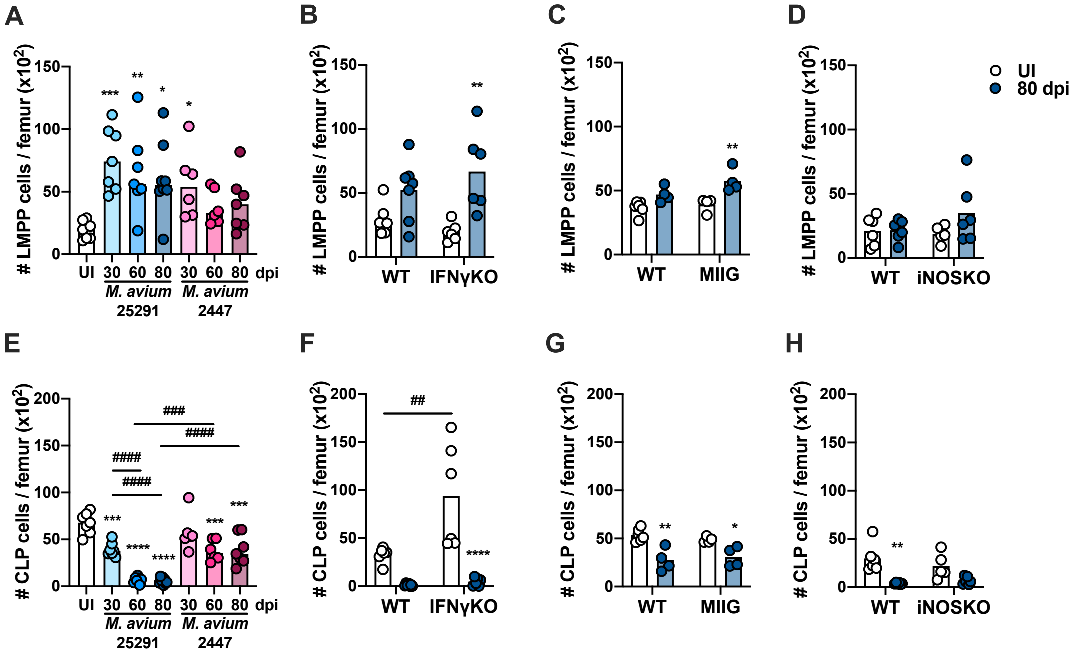

Supplementary Figure 1 | The increase on the number of LMPP and the decrease on the number of CLP cells in mice infected with *M. avium* 25291 is independent on IFNγ and iNOS. **(A)** Number of LMPP cells from uninfected (white) WT mice or infected with *M. avium* strain 25291 (blue) or strain 2447 (pink) at 30, 60 and 80 dpi. **(B, C and D)** Number of LMPP cells from uninfected (white) or infected for 80 days with *M. avium* 25291 (blue), WT, IFNγKO, MIIG or iNOSKO mice. **(E)** Number of CLP cells from uninfected WT mice (white), or infected with *M. avium* strain 25291 (blue) or strain 2447 (pink) at 30, 60 and 80 dpi. **(F, G and H)** Number of CLP cells from uninfected (white) or infected for 80 days with *M. avium* 25291 (blue), WT, IFNγKO, MIIG or iNOSKO mice. Bars represent the mean from 4 to 8 mice per group, from one of two independent experiments. In **(A, E)** comparisons between infected and uninfected mice were evaluated by ordinary one-way ANOVA followed by Dunnett's multiple comparisons test and marked as: * p <0.05, ** p <0.01, *** p <0.001, **** p <0.0001; comparisons between infected groups were evaluated by 2-way ANOVA followed by Tukey's multiple comparisons test and marked as: ### p <0.001, #### p <0.0001. In **(B-D, F-H)**, comparisons were evaluated by 2-way ANOVA followed by Tukey's multiple comparisons test and marked as: * p <0.05, ** p <0.01, **** p <0.0001 for comparisons between uninfected and infected, and as: ## p < 0.01 for comparisons between infected groups. UI stands for uninfected. dpi stands for days post infection.

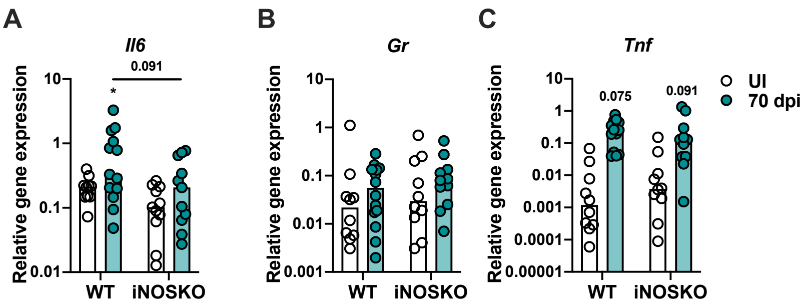

Supplementary Figure 2 | *M. avium* strain 25291 infected iNOSKO mice present lower *Il6* expression levels in the BM. RNA expression levels in the BM of WT and iNOSKO mice uninfected (white) or infected with *M. avium* strain 25291 for 70 days (teal). **(A)** *Il6*, **(B)** *Gr* and **(C)** *Tnf*. Bars represent the median from 10 to 14 mice per group from two pooled independent experiments. Comparisons were performed by 2-way ANOVA followed by Tukey's multiple comparisons test, and marked between uninfected and infected groups as: * p <0.05. For p-values between [0.05; 0.10], their values are represented in the graphs. UI stands for uninfected. dpi stands for days post infection.

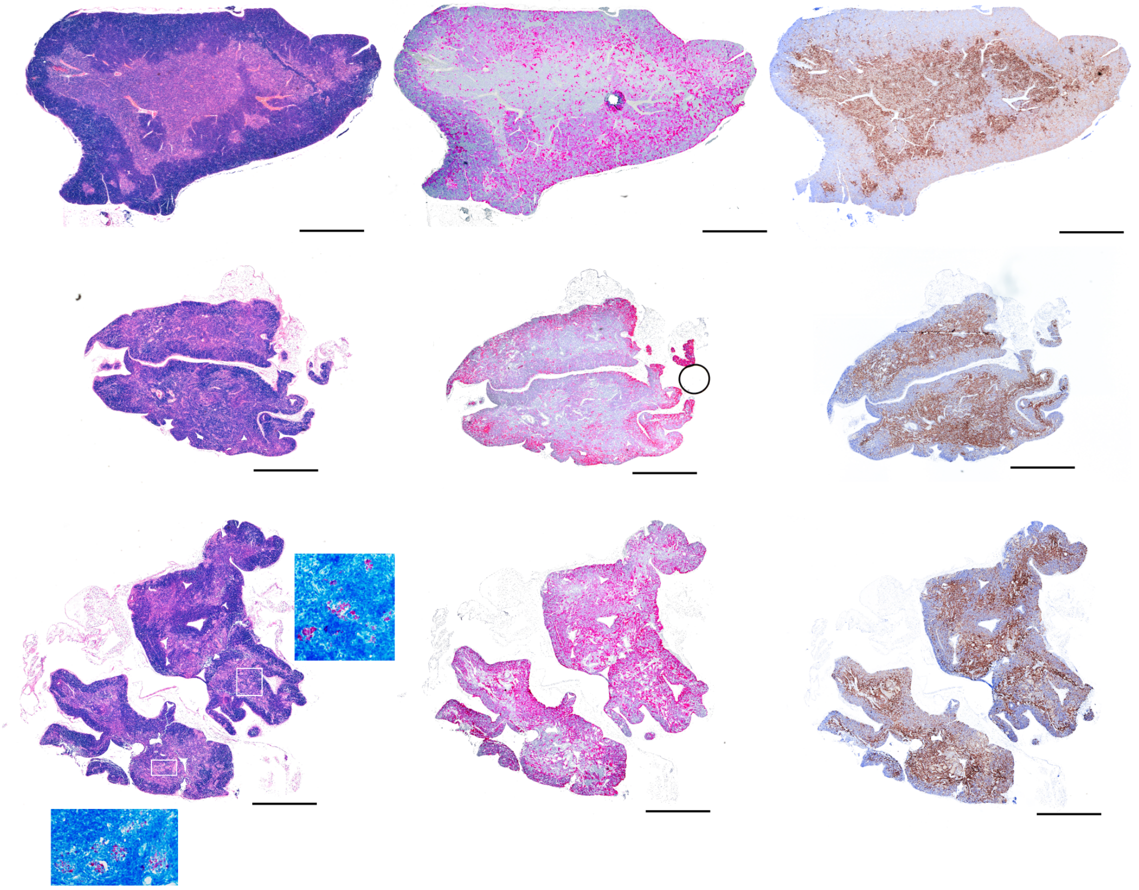

Supplementary Figure 3 | Alterations in the thymus structure from WT mice upon 70 days of infection with *M. avium* strain 25291. Representative hematoxylin-eosin (H&E) stain (left column,) and of immunohistochemistry stains of keratin 8 (K8; middle column) and K5 (right column) of thymi from uninfected (top row) and 70 days infected mice (middle and bottom rows). Insets from the H&E stains depict acid-fast stain by Ziehl-Neelsen. Briefly, thymi were fixed with 4% paraformaldehyde in PBS and embedded in paraffin. Four micrometer serial sections were stained with H&E, Ziehl-Neelsen or immunohistochemistry. Briefly for immunohistochemistry, antigen retrieval was performed using EDTA buffer (1 mM, 0.5% Tween20, pH 8.0; for 30 min at 96 ºC in a water bath, followed by 20 min at room temperature). The primary antibodies anti-K5 (made in rabbit; 1:1000 dilution; Abcam, Cambridge, U.K.) and anti-K8 (clone Troma1; made in rat; 1:50 dilution; developed by P. Brulet and R. Kemler and obtained from the Developmental Studies Hybridoma Bank, developed under the auspices of the National Institute of Child Health and Human Development and maintained by The University of Iowa, Department of Biology, Iowa City, IA) were incubated overnight at 4 ºC. Secondary antibodies used were anti-rat IgG alkaline phosphatase (Vector Labs) or anti-rabbit IgG horseradish peroxidase (Enzo Life Sciences), followed by development with HIGHDEF red IHC chromogen (Enzo Life Sciences) or with DAB substrate (Vector Labs), respectively. Counterstain was performed using 50 % hematoxylin. No significant signal was observed in the negative controls (no antibodies, no primary antibodies, and isotype controls). Slides were visualized using a BX61 microscope with an Olympus DP70 camera. Control of whites and contrast was performed using the Adobe Lightroom Classic. Scale bars represent 1mm.

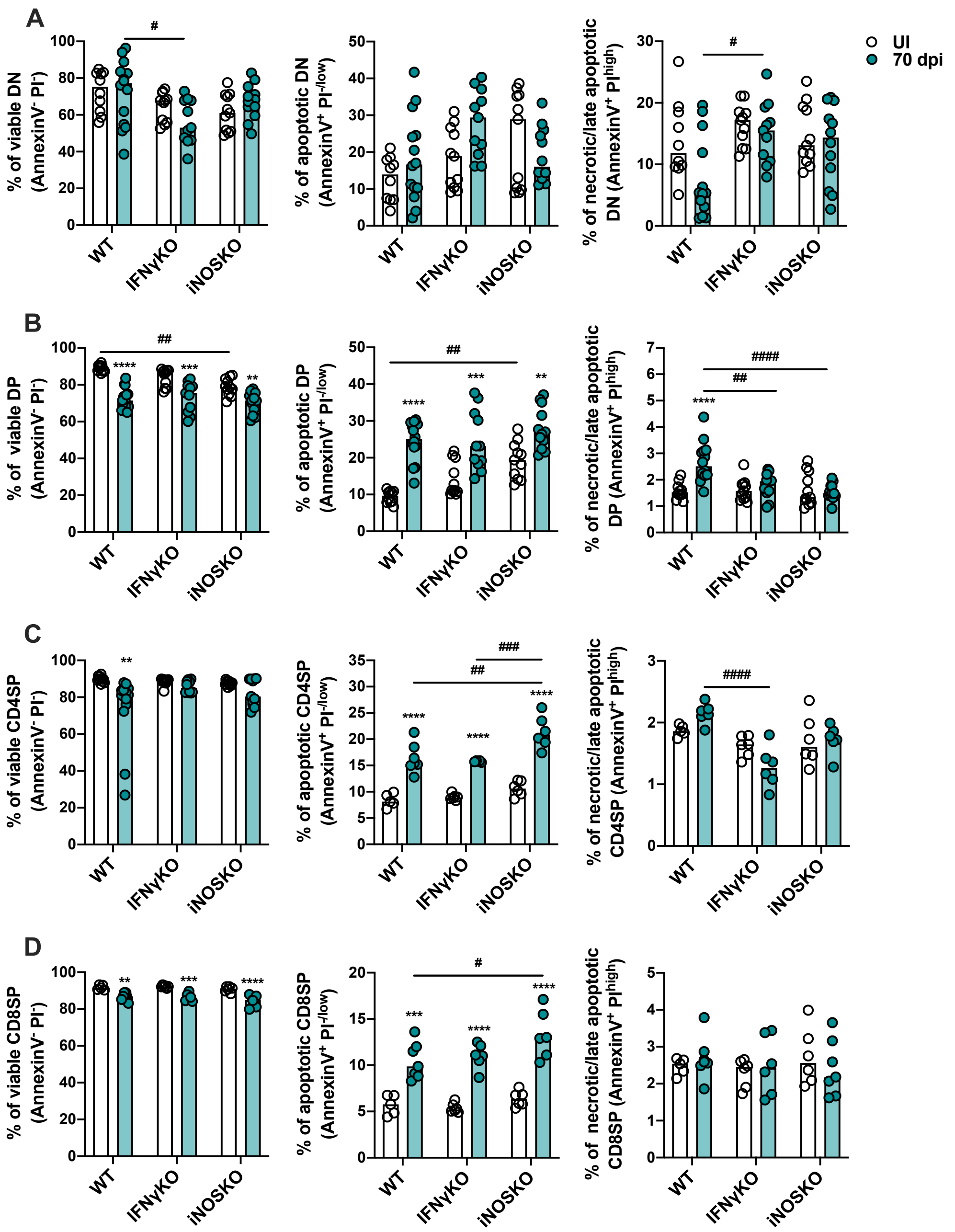

Supplementary Figure 4 | Decreased thymocyte viability after infection is more evident in DP and SP thymocyte populations. Viable (left column – AnnexinV$^-$ PI$^-$), apoptotic (center column – AnnexinV$^+$ PI$^{-/low}$) and necrotic/late apoptotic (right column – AnnexinV$^+$ PI^{high}), DN **(A)**, DP **(B)**, CD4SP **(C)** and CD8SP **(D)** thymocytes from WT, IFNγKO or iNOSKO mice uninfected (white) or infected for 70 days with *M. avium* 25291 (teal). Bars represent the mean from 9 to 14 mice per group from two independent experiments plotted together. Statistically significant differences were accessed by 2-way ANOVA followed by Tukey's multiple comparisons test and marked as ** p <0.01, *** p <0.001, **** p <0.0001 between uninfected and infected, and as # p <0.05, ## p <0.01, ### p <0.001, #### p <0.0001 between infected groups. UI stands for uninfected. dpi stands for days post infection.

Supplementary Table 1 | Genes analysed by RT-qPCR. Primer sequences and respective annealing temperatures.

**Target gene**	**Annealing temperature (ºC)**	**Primer sequence (5' -> 3')**
18S rRNA	58	F: GTA ACC CGT TGA ACC CCA TT<br>R: CCA TCC AAT CGG TAG TAG CG
Ccl21	59	F: CCC TGG ACC CAA GGC AGT<br>R: AGG CTT AGA GTG CTT CCG GG
Ccl25	58	F: GTG CTG TGA GAT TCT ACT TCC<br>R: TAT GGT TTG ACT TCT TCC TTT CAG
Ccr7	59	F: AGA GGC TCA AGA CCA TGA CGG A<br>R: TCC AGG ACT TGG CTT CGC TGT A
Ccr9	58	F: GCC ATG TTC ATC TCC AAC TGC AC<br>R: CCT TCG GAA TCT CTC GCC AAC A
Cxcl12	58	F: CAT CAG TGA CGG TAA ACC AG<br>R: CAC AGT TTG GAG TGT TGA GG
Cxcr4	58	F: GAC TGG CAT AGT CGG CAA TGG A<br>R: CAA AGA GGA GGT CAG CCA CTG A
Gapdh	58	F: GGG CCC ACT TGA AGG GTG GA<br>R: TGG ACT GTG GTC ATG AGC CCT T
Gr	58	F: AGA GCA GTG GAA GGA CAG CAC A<br>R: GCA GCG CGG CAG GAA CTA TT
Hprt	58	F : GCT GGT GAA AAG GAC CTC T<br>R: CAC AGG ACT AGA ACA CCT GC
Ifng	58	F: CAA CAG CAA GGC GAA AAA GG<br>R: GGA CCA CTC GGA TGA GCT CA
Il6	59	F: CCG GAG AGG AGA CTT CAC AG<br>R: TCC ACG ATT TCC CAG AGA AC
iNos	59	F: CTC GGA GGT TCA CCT CAC TGT<br>R: GCT GGA AGC CAC TGA CAC TT
P-selectin	58	F: TCC AGG AAG CTC TGA CGT ACT TG<br>R: GCA GCG TTA GTG AAG ACT CCG TAT
Psgl1	58	F: AGC AGA GCA AGC GTA AGA CGT C<br>R: TCT CCC AGG AAG ATG TTG CGA C
Tnf	58	F: ACT TCG GGG TGA TCG GTC CCC<br>R: GTG GTT TGC TAC GAC GTG GGC TA

Ccl, C-C motif chemokine ligand; Ccr, C-C motif chemokine receptor; Cxcl, C-X-C motif chemokine ligand; Cxcr, C-X-C motif chemokine receptor; F, forward; *Gapdh*, Glyceraldehyde 3-phosphate dehydrogenase; *Gr*, glucocorticoid receptor; *Hprt*, hypoxanthine guanine phosphoribosyl transferase; *Ifng*, interferon gamma; *Il6*, interleukin-6; iNos, inducible nitric oxide synthase; Psgl1, P-selectin glycoprotein ligand-1; R, reverse; *Tnf*, tumor necrosis factor.
